# Association between trauma exposure and respiratory disease-A Mendelian randomization study

**DOI:** 10.3389/fendo.2022.1001223

**Published:** 2022-09-05

**Authors:** Yuchao Ma, Changjiang Meng, Liang Weng

**Affiliations:** ^1^ Department of Cardiothoracic Surgery, Third Xiangya Hospital, Central South University, Changsha, China; ^2^ Clinical Research Center, Third Xiangya Hospital, Central South University, Changsha, China; ^3^ Xiangya Cancer Center, Xiangya Hospital, Central South University, Changsha, China; ^4^ Key Laboratory of Molecular Radiation Oncology Hunan Province, Xiangya Hospital, Central South University, Changsha, China; ^5^ Hunan International Science and Technology Collaboration Base of Precision Medicine for Cancer, Xiangya Hospital, Central South University, Changsha, China; ^6^ Institute of Gerontological Cancer Research, National Clinical Research Center for Gerontology, Changsha, China; ^7^ Xiangya Lung Cancer Center, Xiangya Hospital, Central South University, Changsha, China

**Keywords:** trauma, respiratory disease, Mendelian randomization, MVMR, instrumental variable (IV)

## Abstract

**Background:**

Trauma is a well-known risk factor for many disease, but the effect of trauma on respiratory disease is unclarified. In the present study, we aimed to evaluate the association between trauma and respiratory disease.

**Method:**

Using both United Kingdom biobank and Finnish biobank genome-wide association study data (GWAS), we performed a two-sample Mendelian randomization (MR) analysis to evaluate the relationship between trauma and respiratory disease. We used four methods including inverse-variance weighted (IVW), weighted median, Maximum likelihood, and MR-Egger in this MR analysis. The IVW MR was selected as the main method. We also performed multivariable Mendelian randomization (MVMR) to simultaneously assess the independent impact of trauma exposure on respiratory disease.

**Results:**

In the main two-sample MR analysis, trauma exposure was significantly associated with increased risk of respiratory disease (OR 1.15, 95%CI: 1.05-1.25). Besides, there was no heterogeneity and horizontal pleiotropy observed in the sensitivity analysis. After adjusting for pack years of smoking and body mass index (BMI), trauma exposure retained its association with respiratory disease (OR, 1.13, 95%CI, 1.04-1.23 adjusted by pack years of smoking; and OR, 1.11, 95%CI, 1.04-1.18 adjusted by BMI).

**Conclusion:**

Our study discovered the association between trauma exposure and the increased risk of respiratory disease, suggesting the prevention and treatment with trauma to reduce the risk of respiratory disease.

## Introduction

Trauma is a public health problem worldwide and a leading cause of mortality. According to the report from World Health Organization (WHO) (1), the number of deaths due to trauma is about 10% of the world’s deaths each year, which is 5.8 million people. Thus, the socio-economic and health burden of trauma needs to be given more attention.

Previous studies have confirmed that trauma is a risk factor for many diseases including cardiovascular disease, respiratory disease, infectious diseases, sepsis, shock, and so on (2–5). Notably, the respiratory disease caused by trauma is very severe, such as acute respiratory distress syndrome (ARDS). A study conducted by Rubenfeld Gordon D. et al. found that 7% of ARDS cases are attributed to trauma, and the mortality rate for trauma-related ARDS is assessed at 24% (6). It is generally considered that trauma can promote a series of inflammatory responses and oxidative stress, leading to diverse diseases (7).

While a few cross-sectional or cohort studies have investigated the association between trauma and respiratory disease (8), these observational studies are susceptible to a variety of confounding factors. Mendelian randomization (MR) is a genetic instrumental variable analysis, that assesses the underlying association between exposure and outcome (9), which is less susceptible to confusion and reverses causal bias (10). Therefore, in the present study, we performed the MR analysis to evaluate the effect of trauma exposure on the risk of respiratory diseases.

## Method

**Figure 1:**
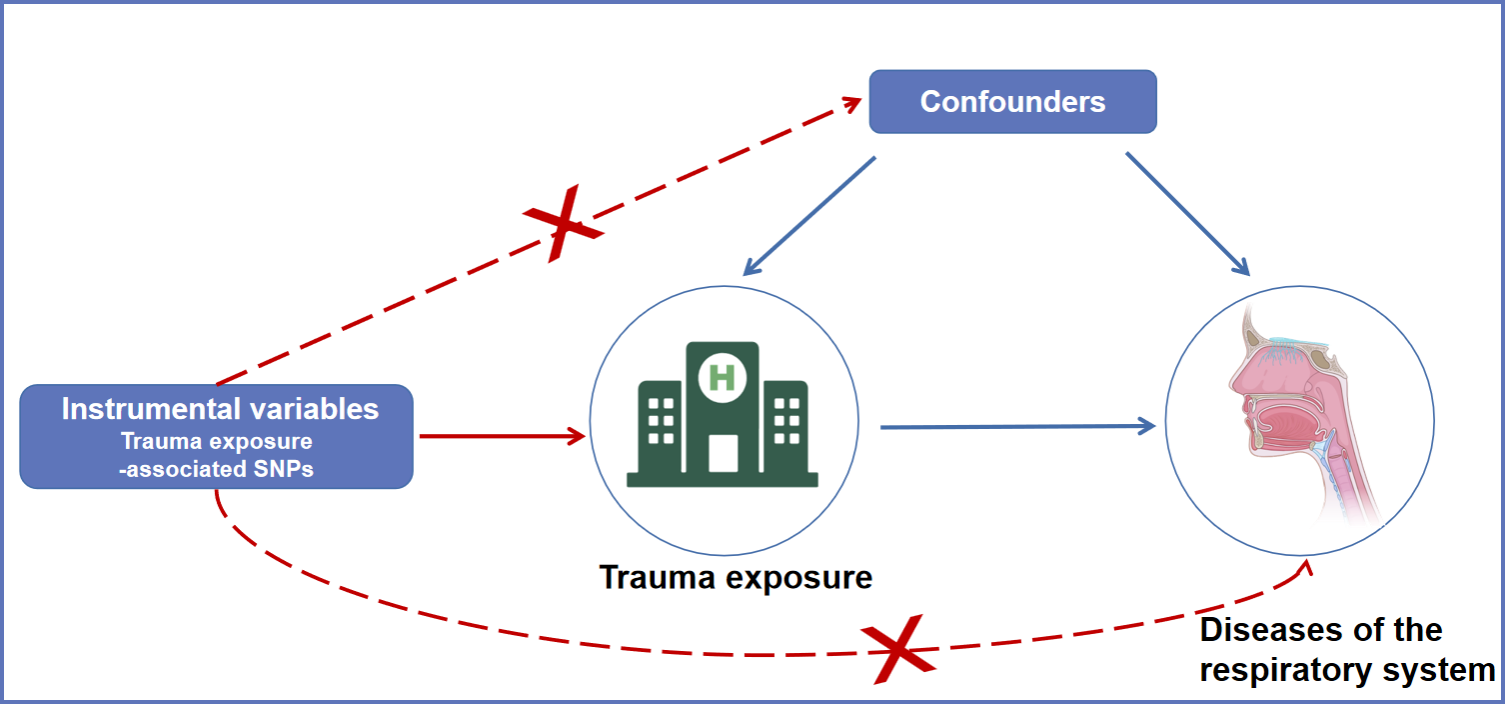
An overview of the MR assumptions. SNPs, single nucleotide polymorphisms; MR, Mendelian randomization.

The overview design of the current MR study is shown in **Figure 1**. The causal relationships between trauma exposure and diseases of the respiratory system risk were explored by a two-sample MR analysis. The valid MR analysis needs to satisfy thise three assumptions: first, the instrument Single-nucleotide polymorphisms (SNPs) are associated with the exposure (trauma exposure); second, the instrument SNPs are not associated with confounding; and third, the instrument SNP influences the outcome *via* the exposure.

### Instrumental variable selection

Genetic instruments for “trauma exposure” were identified from the United Kingdom Biobank (UKB) (11). The study included 98,720 subjects, with 35,269 cases and 63,451 controls. The instrument SNP was filtered based on the threshold of P < 5 × 10^-8^. F statistic represents the strength of the relationship between SNPs and exposures. F statistic related to the explained variance for exposure (R^2^), sample size (n) and number of SNPs (k) by the formula F = [(n−k−1)/k]/[R^2^/(1−R^2^)]. Generally, F>10 indicates that selected SNPs strongly predict trauma.

Pack years of smoking data were also extracted from the UKB. Pack years calculated for individuals who have ever smoked. A pack year was defined as the number of cigarettes smoked per day, divided by twenty, multiplied by the number of years of smoking: pack years=Number of cigarettes per day/20*(Age stopped smoking - Age start smoking). If the individuals who gave up smoking for more than 6 months, the figure will be adjusted, and calculated as follow: pack years = Number of cigarettes per day/20*(Age stopped smoking - Age start smoking - 0.5). The BMI summary data were extracted from a large meta-analysis of GWASs conducted by the GIANT consortium, enrolling 125 GWASs in more than 339,224 individuals (12). In this study, we limited our selection of SNPs to European ancestry (n = 322,154) (**Table S1** **).**

### Outcome data sources

We extracted GWAS summary data of diseases of the respiratory system (case/control=107,261/111,531; overall=218,792) from MR-base website (http://app.mrbase.org/).

### Power calculations

We used a non-centrality parameter-based approach to evaluate the power of our study on a publicly available mRnd web tool (http://cnsgenomics.com/shiny/mRnd/). For the binary outcome (diseases of the respiratory system) in this MR study, after we imputed the required parameters in mRnd (α=0.05 in this study), the minimum detectable OR was roughly estimated. The results of power calculations are shown in **Table S2**.

### Statistical analysis

The associations between exposure (trauma exposure) and outcome (diseases of the respiratory system) were calculated with a two-sample MR analysis using the inverse variance weighted (IVW) method as the primary causal effect estimates to explore the causal associations. The results were shown as odds ratios (OR) and 95% confidence intervals (CI).

### Sensitivity analysis

**Figure 2:**
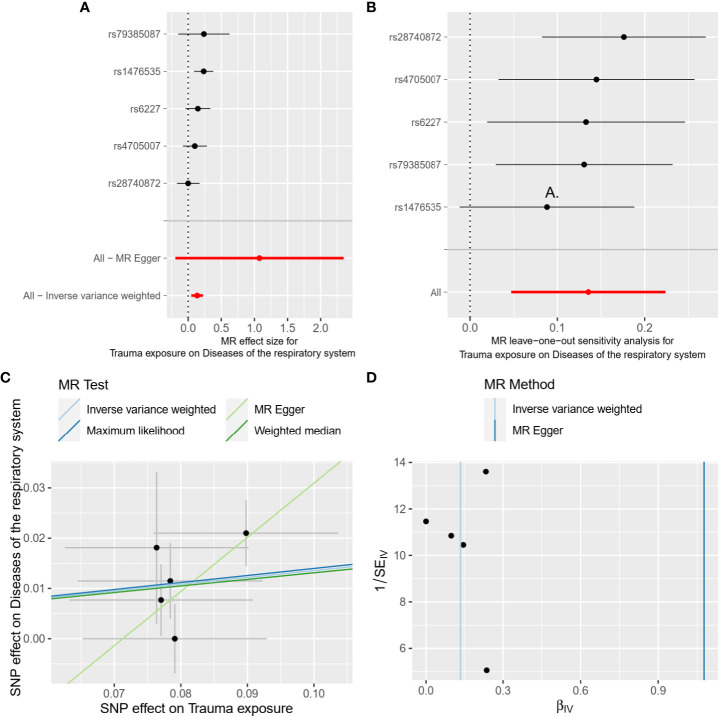
Forest plot **(A)**, leave-one-out analysis **(B)**, scatter plot **(C)**, and funnel plot **(D)** of the causal effect of trauma exposure on diseases of the respiratory system risk. MR, Mendelian randomization; SNPs, single nucleotide polymorphisms.

We performed some sensitivity analysis using other methods including MR Egger, Weighted median, and Maximum likelihood to consider the consistency across all MR methods. To test the robustness of the results, we also performed the leave-one-out analysis using the IVW method (**Figure 2B**). Moreover, we used the heterogeneity test and Egger regression intercept to examine the reliability of MR estimates. Considering the effects of common risk factors such as smoking and body mass index (BMI) on the respiratory system, multivariable Mendelian randomization (MVMR) was also conducted to estimate the effect of trauma after adjusting for pack years of smoking and BMI. In this study, MR-PRESSO global test were conducted to examine the reliability of the results based on instrumental SNP extracted from trauma exposure.

P value < 0.05 was thought to be statistically significant. All analyses were performed in R (version 4.0.3) with R packages “TwoSampleMR”, “MVMR” and “MR-PRESSO”.

## Result

### Character of SNP for analysis

**Table 1** shows the source of GWAS summary data for exposure and outcome. Each SNP extracted from trauma exposure with P-value < 5×10^-8^ and its F statistic and R^2^ were presented in **Table 2**. There were 5 instrument SNPs selected for trauma with F statistics ranging from 30 to 42, representing that the MR estimates calculated based on these SNPs are reliable and do not lead to bias from weak instruments.

**Table 1:** Characteristics of trauma exposure and diseases of the respiratory system datasets.

Exposure	Consortium	SNP	Cases/Controls	Sample size	Population
trauma exposure	Uk biobank	5	35,269/63,451	98,720	European
**Outcome**	**Data source**	**Studies**	**Cases/Controls**	**Sample size**	**Population**
diseases of the respiratory system	FINN biobank	–	107,261/111,531	218,792	European

SNPs, single nucleotide polymorphisms.

**Table 2:** Single-nucleotide polymorphisms associated with trauma exposure.

CHR	SNP	SE	P-value	BETA	Samplesize	effect_allele	other_allele	EAF	F
5	rs4705007	0.0137786	2.25E-08	-0.0770422	98720	A	G	0.657323	31.3
7	rs1476535	0.0138727	9.55E-11	0.0898103	98720	T	C	0.551865	41.9
14	rs28740872	0.0138576	1.14E-08	0.0790989	98720	T	A	0.373132	32.6
15	rs6227	0.0138922	1.65E-08	-0.0784234	98720	T	C	0.329555	31.9
16	rs79385087	0.0137777	2.97E-08	-0.076375	98720	T	C	0.047415	30.7

SNP, single nucleotide polymorphisms.

### Association between trauma exposure and diseases of the respiratory system

In the main analysis, we found there was evidence for a strong causal association between trauma exposure and disease of the respiratory system (OR, 1.15, 95%CI, 1.05-1.25 for the IVW method, **Table 3**). The results of weighted median and Maximum likelihood were consistent with IVW (OR, 1.14, 95%CI, 1.02-1.27 for the weighted median method; OR, 1.15, 95%CI, 1.05-1.25 for the maximum likelihood method, **Table 3**). However, the results of MR Egger showed that there was no statistically significant between trauma exposure and diseases of the respiratory system (OR, 2.94, 95%CI, 0.82-10.47 for MR Egger method, **Table 3**), but the direction was consistent with the results of the main analysis. In the MVMR analysis (**Table 3**), trauma exposure still showed an adverse effect on respiratory system disease after adjusting for pack years of smoking (OR, 1.13, 95%CI, 1.04-1.23) and BMI (OR, 1.11, 95%CI, 1.04-1.18), respectively. According to the results of the statistical power at the given OR, the association between trauma exposure and diseases of the respiratory system was robust. The forest plot of a single SNP was shown in **Figure 2A**.

**Table 3:** Two-sample Mendelian randomization estimations showing the effect of trauma exposure on the risk of diseases of the respiratory system.

Outcomes	Methods	Odds ratio (95% CI)	*P*-value	Q-statistics	*P*_h_	Egger intercept	*P_intercept_*
**Diseases of the respiratory system**	MR Egger	2.94 (0.82-10.47)	0.195	2.51	0.473	-0.08	0.242
	Weighted median	1.14 (1.02-1.27)	0.019				
	Inverse variance weighted	1.15 (1.05-1.25)	0.003	4.63	0.327		
	Maximum likelihood	1.15 (1.05-1.25)	0.002				
	MVMR adjusted by Pack years of smoking	1.13 (1.04-1.23)	0.004				
	MVMR adjusted by body mass index	1.11 (1.04-1.18)	0.002				

MR, Mendelian randomization; MVMR, multivariable Mendelian randomization.

The results of heterogeneity were not observed in overall MR results (all P_h_ ≥ 0.05, **Table 3**). Furthermore, horizontal pleiotropy was not found in overall MR results (all Pegger_intercept ≥ 0.05, **Table 3**). The scatter plot and funnel plot were presented in **Figure 2C, D**, respectively, showing the consistency of the results. The above results all suggested that the results in our analysis were convincing. The detailed results for the heterogeneity test and pleiotropy test were shown in **Table 3**. Moreover, the global test of MR-PRESSO analysis proved that there was no horizontal pleiotropy in our analysis between trauma exposure and diseases of the respiratory system (P = 0.360), and no outliers were found (**Table S3**).

## Discussion

In this two-sample MR analysis using large sample GWAS data, we observed that the trauma expsoure will contribute to the risk of disease of respiratory systems, which indicated the care and treatment for the respiratory systems after trauma exposure.

Trauma usually refers to injury in four components, including physical, sexual, emotional abuse and general trauma (2). Systemic responses, such as hemostasis, inflammation, endocrine, and neurological interactions, are involved in severe trauma (13). Numerous modifications to the body’s immunological, endocrine, and metabolic pathways take place after trauma. The complement system is triggered by trauma-induced tissue injury, which causes neutrophils and macrophages to become active and release inflammatory mediators such as interleukin-1, tumor necrosis factor (TNF), and platelet-activating factor (14). The excessive infiltration and activation of neutrophils in respiratory system cause tissue injury, which may be caused by neutrophil extracellular traps (NETs) (15). Clinical research has demonstrated an association between the onset of chronic respiratory disease and local and systemic neutrophil increase. Additionally, research on animals has demonstrated that airway-infiltrating neutrophils have an impoertant role in both the beginning and continuation of airway inflammation (16).

Interleukin 1 receptor 1 (IL-1R1) signaling is critical for lung fibrosis and inflammation of mice (17). Transforming growth factor β (TGFβ) is a key pro-fibrotic cytokine, which is augmented by IL-1. Elevated levels of TGFβ activate the proliferation of epithelial cells and fibroblasts and their conversion to myofibroblasts, ultimately leading to pulmonary fibrosis (18, 19). TNFα levels are elevated in a variety of inflammatory lung diseases including asthma, chronic obstructive pulmonary disease, acute lung injury, acute respiratory distress syndrome, nodal disease, and interstitial lung disease pulmonary fibrosis. TNFα induces the formation of inflammatory cells, promotes the generation of inflammatory mediators, and causes oxidative and nitrosative stress, airway hyperreactivity, and tissue remodeling, among other pathological effects (20). Patients with non-small cell lung cancer were shown to have considerably higher expression levels of IL-1 and TNFα (21). In terms of immune response pathways, these findings help to partially explain the higher risk of respiratory disease following trauma exposure.

The most frequent respiratory diseases brought on by trauma are pneumothorax and ARDS. Pneumothorax can occur in up to 50% of people who have had severe multiple chest injuries (22). Traumatic pneumothorax is the presence of air between the visceral and mural pleura as a result of penetrating and non-penetrating (blunt) traumatic events, with ongoing lung retraction from the chest wall (23). In addition to pneumothorax, studies have shown that major trauma, including blunt force trauma, penetrating injuries, and burns predispose to ARDS (24). The pathophysiology of trauma-induced ARDS is complicated by several causes, of which cellular damage, the breakdown of cellular junctions, and disruption of cellular barriers lead to increased vascular permeability and greater air-blood distances are key players. First, trauma induces direct harm to epithelial and endothelial lung cells and prompts the release of endogenous danger-associated molecular patterns (DAMP). DAMP is capable of inducing pulmonary and systemic inflammatory responses, which in turn induce epithelial and endothelial lung injury *via* excessive immune responses. Additionally, the accompanying pro-inflammatory environment can have a deleterious impact on cell adhesion junctions (25). Involved in this process are both the activation of the complement system pathway and the synthesis of C5a by lipopolysaccharide (26).

Using data from a large estabished sample of GWAS data, our study systematically evaluated the association between trauma exposure and the chance of acquiring the respiratory disease. To our knowledge, this study is the first study to use MR analysis to investigate the causal association between trauma and respiratory disease. Our findings imply that suitable steps can be taken to prevent the development of respiratory diseases following trauma exposure. There are several limitations to this study. First, the sample relies too heavily on individual European pedigrees, limiting generalizability to other pedigrees. Second, the trauma exposure in this study was determined according to self-reported questionnaires. Thus, it should be cautious when considering our results. Third, our study explored only whether the liability to trauma exposure is associated with disease of respiratory system, so the causal pathways leading to trauma exposure that causes disease of respiratory system could not be ruled out.

## Conclusion

In conclusion, this study corroborates the data that trauma exposure is related to an increased risk of respiratory disease, highlighting the significance of respiratory care following the onset of trauma.

## Data availability statement

Publicly available datasets were analyzed in this study. The datasets were derived from sources in the public domain: UK biobank (https://www.ukbiobank.ac.uk/) and MR-Base (https://www.mrbase.org/).

## Ethics statement

This study is a secondary analysis conducted through existing GWAS data and UK biobank. The specific ethics and consent statements reviewed in this study can be accessed in the original publication.

## Author contributions

All authors participated in the field survey and data collection. YM and LW drafted the manuscript. YM and CM analyzed the data. LW designed the study. YM and LW obtained the funding. All authors participated in the field survey and data collection, critically revised the manuscript, and gave final approval to the version submitted for publication.

## Funding

National Multidisciplinary Cooperative Diagnosis and Treatment Capacity Building Project for Major Diseases (Lung Cancer, Z027002), National Natural Science Foundations of China (81974465 and 81900199), the recruitment program for Huxiang Talents (2019RS1009) received by L. And Scientific Research Project of Hunan Provincial Health Commission (202204024507) received by YM.

## Conflict of interest

The authors declare that the research was conducted in the absence of any commercial or financial relationships that could be construed as a potential conflict of interest.

## Publisher’s note

All claims expressed in this article are solely those of the authors and do not necessarily represent those of their affiliated organizations, or those of the publisher, the editors and the reviewers. Any product that may be evaluated in this article, or claim that may be made by its manufacturer, is not guaranteed or endorsed by the publisher.

## Supplementary material

Supplementary Table 1 Description of public GWAS data used for in MVMR.

Supplementary Table 2 Statistical power calculation for Mendelian randomization analyses.

Supplementary Table 3 Application of MR-PRESSO to detect horizontal pleiotropy in Mendelian randomization analysis.

## Abbreviations

WHO, World Health Organization; MR, Mendelian randomization; GWAS, genome-wide association study; MVMR, multivariable Mendelian randomization; ARDS, acute respiratory distress syndrome; BMI, body mass index; IVW, inverse-variance weighted; MR-PRESSO, MR-Pleiotropy Residual Sum and Outlier; OR, odds ratio; CI, confidence intervals; IV, instrumental variable; SNP, Single-nucleotide polymorphism; LD, linkage disequilibrium; TGFβ, Transforming growth factor β; NETs, neutrophil extracellular traps; DAMP, danger-associated molecular patterns.
