# Supplementary material for: Association between trauma exposure and respiratory disease-A Mendelian randomization study
Source: Front Endocrinol (Lausanne). 2022 Sep 5;13:1001223. doi: 10.3389/fendo.2022.1001223 (PMC9483852; doi:10.3389/fendo.2022.1001223)
Supplement: Supplementary Table 1 — Description of public GWAS data used for in MVMR. [file DataSheet_1.docx]

**Supplementary Materials**

**Table S1. Description of public GWAS data used for in MVMR.**

| **Exposure** | **Consortium** | **Sample Size** | **Population** | **Sex** |
| --- | --- | --- | --- | --- |
| Pack years of Smoking | UK biobank | 142387 | European | Males and females |
| Body mass index | GWAS | 322154 | European | Males and females |

GWAS: genome-wide association study; MVMR: multivariable Mendelian randomization.

**Table S2. Statistical power calculation for Mendelian randomization analyses.**

| **Statistical power at the given odds ratio** | | | | | | | | | | |
| --- | --- | --- | --- | --- | --- | --- | --- | --- | --- | --- |
| **Exposure** | **Outcome** | **Sample size** | **Cases** | **OR = 0.10** | **OR = 0.50** | **OR = 0.80** | **OR = 1.00** | **OR = 1.20** | **OR = 1.50** | **OR = 2.00** |
| Trauma-5 SNPs | Diseases of the respiratory system | 218,792 | 107,261 | 1.00 | 1.00 | 1.00 | 0.05 | 1.00 | 1.00 | 1.00 |

OR: Odds ratio

Table S3. Application of MR-PRESSO to detect horizontal pleiotropy in Mendelian randomization analysis.

| **Outcomes** | **Exposure** | **Outliers** | **Global Test-P value** |
| --- | --- | --- | --- |
| Trauma | Respiratory system disease | None | 0.36 |
